# Planetary Gearbox Dynamic Modeling Considering Bearing Clearance and Sun Gear Tooth Crack †

**DOI:** 10.3390/s21082638

**Published:** 2021-04-09

**Authors:** Xianhua Chen, Xingkai Yang, Ming J. Zuo, Zhigang Tian

**Affiliations:** Department of Mechanical Engineering, University of Alberta, Edmonton, AB T6G 1H9, Canada; xianhua@ualberta.ca (X.C.); xingkai2@ualberta.ca (X.Y.); ming.zuo@ualberta.ca (M.J.Z.)

**Keywords:** dynamic modeling, planetary gearbox, bearing clearance, sun gear tooth crack

## Abstract

Planetary gearbox systems are critical mechanical components in heavy machinery such as wind turbines. They may suffer from various failure modes, due to the harsh working environment. Dynamic modeling is a useful method to support early fault detection for enhancing reliability and reducing maintenance costs. However, reported studies have not considered the sun gear tooth crack and bearing clearance simultaneously to analyze their combined effect on vibration characteristics of planetary gearboxes. In this paper, a dynamic model is developed for planetary gearboxes considering the clearance of planet gear, sun gear, and carrier bearings, as well as sun gear tooth crack levels. Bearing forces are calculated considering bearing clearance, and the dynamic model equations are updated accordingly. The results reveal that the combination of bearing clearances can affect the vibration response with sun gear tooth crack by increasing the kurtosis. It is found that the effect of planet gear bearing clearance is very small, while the sun gear and carrier bearing clearance has clear impact on the vibration responses. These findings suggest that the incorporation of bearing clearance is important for planetary gearbox dynamic modeling.

## 1. Introduction

Mechanical components play critical roles in many engineering systems, such as railway systems, trucks, automobiles, and conveyor belts. For example, bearings are used to provide the needed support to rotating shafts. Gearboxes are often needed to regulate speed and torque for the target applications. For the gear set in a planetary gearbox, many studies analyzed its failure such as tooth crack focusing on condition monitoring [1,2,3,4,5], signal processing [6,7], and machine learning methods [8,9] that utilize condition monitoring data such as vibration signals to evaluate the condition of health for the running mechanical systems. To develop condition monitoring tools for reliability assurance of mechanical systems, researchers have analyzed machine dynamics [10], seeded faults into laboratory mechanical systems [11], developed effective signal processing methods [12], and utilized degradation prediction approaches [13].

**Figure 1:**
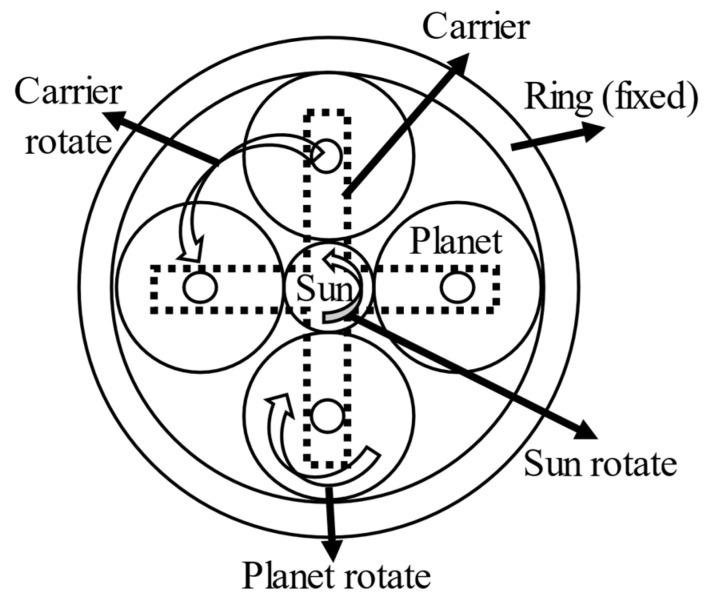
The structure of planetary gearbox [14].

Planetary gearboxes are important components in many applications, such as automobiles, mining machines, and wind turbines. The major rule of the planetary gearbox is to increase or reduce the input speed. For example, the transmission uses planetary gearboxes to change the speed of cars. Figure 1 shows the schematic of a planetary gearbox with four planet gears. According to the complicated structure, the planetary gearboxes have large load capacity and are widely used in a high-load environment. Although well-designed, various failure modes such as gear faults in gearboxes may destroy the planetary gearboxes. If no early detection, these faults could cause large economic loss or catastrophe.

Some planetary gearboxes operate in high load operating environments [4,7], while some operate under relatively low load [15,16]. In harsh conditions under high load, gearboxes may suffer considerable fault modes [17], and about 60% of faults are caused in the gear tooth, such as the sun-gear tooth crack [18]. In this paper, the sun gear contacts four planet gears. Chaari et al. [19] pointed out that the tooth damage can be increased during the gear meshing. When the sun gear is the input component loading the torque and meshing with planet gears, it is important to do the early detection to prevent gearbox failures. The tooth crack can affect the dynamic responses by decreasing the mesh stiffness [20]. Furthermore, mesh stiffness is one of the important parameters in dynamic modeling. According to the mesh stiffness, researchers can simulate the vibration responses of the planetary gearboxes to make the fault diagnosis [20,21,22].

**Figure 2:**
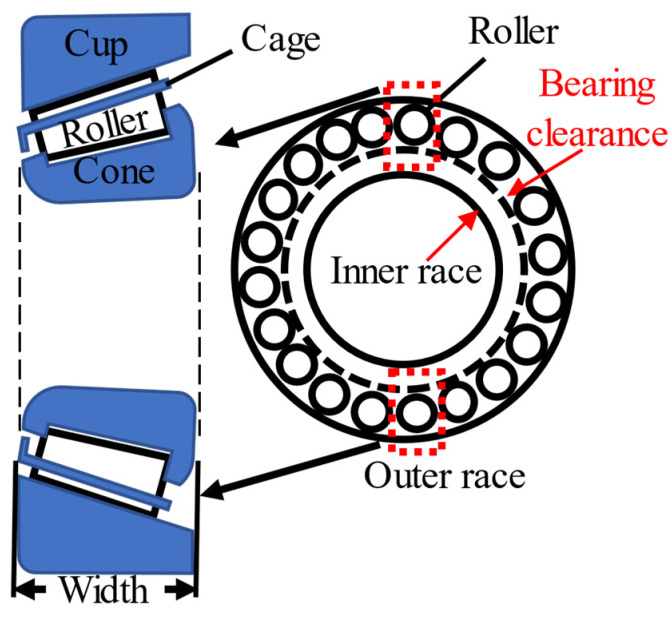
Bearing with clearance [21].

In the planetary gearbox, bearings are also important components, except the gears. In this paper, the planetary gearbox uses tapered roller bearings, and the structure is shown in Figure 2. The rollers’ location is between the inner and outer race. Then, all the rollers are held in a cage. According to this figure, the rollers may not be simultaneously in contact with both the outer race and the inner race. This means that there may be a gap before the inner race gets in touch with a roller, which in turn gets in touch with the outer race. This gap is called the bearing clearance, which can affect the bearing stiffness [23,24].

The bearing clearance consideration results in a nonlinear dynamic problem [25]. The linear system means the relationship between the bearing force and gear displacement is linear, while the nonlinear system shows that this relationship is not linear. The dynamic modeling with bearing clearances has been considered in dynamic modeling. For example, the load of a planetary gearbox with bearing clearance was studied [26]. The tooth wedging in a planetary gearbox with bearing clearances were also analyzed [24]. According to the previous studies, the bearing clearance can affect the planetary gearboxes in the helicopter [27] or the wind turbine [23]. Although previous works have studied either the bearing clearance [28] or sun gear tooth crack for planetary gearbox dynamic modeling [29], the combined effects of bearing clearance and sun gear tooth crack have not been considered in the reported studies. As mentioned above, the bearing clearance and sun gear tooth crack both can affect the dynamic responses, and thus this paper focuses on these two parameters.

Furthermore, a planetary gearbox dynamic model considering more details of gearbox structure, such as different locations of bearings, could be more authentic. In terms of bearing locations, there are sun gear bearings, ring gear bearings, planet gear bearings, and carrier bearings. There may also be a special bearing called ring-carrier bearing, which is analyzed in Reference [28]. However, the real planetary gearbox in this paper does not have the ring-carrier bearing. In addition, the ring gear is fixed in this paper, and thus there are only sun gear, planet gear, and carrier bearings.

This paper considers the clearance of planet gear, sun gear, and carrier bearings, as well as sun gear tooth crack levels, to simulate the planetary gearbox. There are 3 different types of bearing clearances, including the sun gear, planet gear, and carrier bearing clearance. According to the analysis of the effect of different types of bearing clearance, the proposed planetary gearbox dynamic model in this paper generates 5 new types of dynamic responses. The first type is the simulated signal with only carrier bearing clearance considered, the second is the dynamic response with only sun gear bearing clearance considered, the third is the simulated response with only planet gear bearing clearance considered, the fourth is the signal with sun gear and carrier bearing clearance considered, and the fifth is the response with sun gear, carrier, and planet gear bearing clearance considered.

For the planetary gearbox, there are two common coordinate systems. The first coordinate system is fixed to the ground and we will call it the fixed coordinate system. The second coordinate system is fixed to the carrier. Since the carrier rotates as the system operates, we will call the second coordinate system the rotating coordinate system. The fixed coordinate system is suitable to analyze the response signals, which is used in Reference [30], while the rotating coordinate system is suitable for formulating motion equations, which is used in Reference [29]. This study uses the rotating coordinate system to build the motion equations for planetary gearbox, while the dynamic responses are analyzed in the fixed coordinate system, which can be derived by conducting coordinate transform to the rotating coordinate system.

In summary, many researchers only considered the bearing clearance, or only considered the sun-gear tooth crack in planetary gearbox dynamic modeling. This paper combines the bearing clearance and sun-gear tooth crack together, and analyzes the effect of sun-gear, planet-gear, and carrier bearing clearance on vibration responses. Thus, six cases with different combinations of bearing-clearance types are investigated to analyze the effect of different combinations of bearing clearance. Case 1 is the dynamic modeling without any bearing clearance, Cases 2 to 4 simulate the vibration response with only one type of bearing clearance, Case 5 considers the combination of the sun gear and carrier bearing clearance, and Case 6 corresponds to the combination of all types of bearing clearance.

The remainder of this paper is organized as follows. In Section 2, the proposed methodology is introduced in detail. Section 3 presents the analysis results of the planetary gearbox dynamic responses together with the related discussions. The conclusions are made in Section 4.

## 2. Proposed Methodology

### 2.1. Planetary Gearbox System

A single-stage planetary gearbox with the ring gear fixed and with four planet gears is considered in this study. All rotating components are supported by bearings. Thus, there are bearings for the sun gear, for each of the planet gears, and for the carrier. This planetary gearbox dynamic model considered in this paper is developed base on the real system discussed in this section.

**Figure 3:**
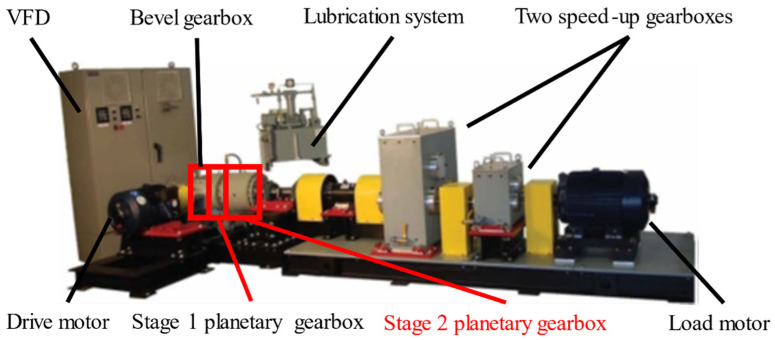
Planetary gearbox test rig [14].

The planetary gearbox system considered in this study is the same as the system in References [29,30], hosted at the Reliability Research Lab in the Department of Mechanical Engineering at the University of Alberta, Edmonton, Canada, shown in Figure 3. The variable-frequency drive (VFD) is used to control the test rig. The drive motor can simulate the high-load environment. Furthermore, the lubrication system reduces the friction between gears. In this test rig, there are five gearboxes, including one bevel gearbox, two planetary gear sets, and two speed-up gearboxes. It should be noted that the Stage 2 planetary gearbox shown in Figure 3 will be employed as a research target in this work. The detailed parameters of the bevel and planetary gearboxes including the number of teeth and their reduction ratios are shown in Table 1. There are three planet gears in the first-stage planetary gear set, while four planet gears are in the second-stage planetary gear set.

**Figure 4:**
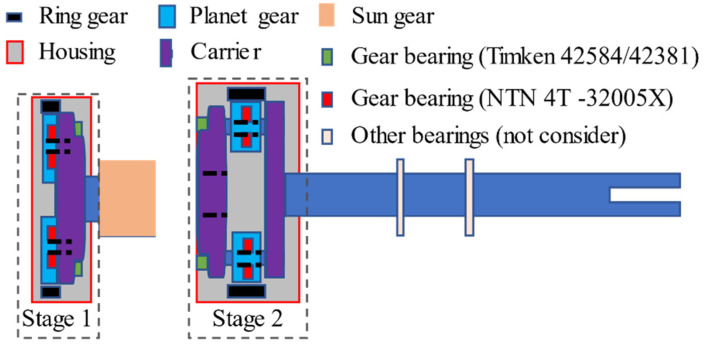
The structure of Stages 1 and 2 planetary gearboxes [14].

Figure 4 illustrates that the carrier of Stage 1 is connected with the sun gear of Stage 2. Furthermore, in Stage 1, there are three identical planet gears and their corresponding bearings, one carrier and its corresponding bearing, and one ring gear without bearing. In Stage 2, there are four identical planet gears and their corresponding bearings, one carrier and its corresponding bearing, and one fixed ring gear without bearing. The output shaft bearings are not considered in this paper, and only the planetary gearbox in Stage 2 is focused on.

Based on the discussions above, the Stage 1 carrier and Stage 2 sun gear use the same bearings. Therefore, the Stage 2 planetary gearbox has three different bearing-clearance types, including carrier bearing (Stage 1), carrier bearing (Stage 2), and four planet gear bearings. The parameters of these bearings in the two-stage planetary gearboxes are listed in Table 2. The parameters ID and OD are the bearing inner and outer race diameters, respectively.

According to the parameters of bearing, the bearing clearance is the same as the clearance in Reference [14]. The results of the bearing clearance are 0.080 mm sun gear bearing, 0.080 mm carrier bearing, and 0.035 mm planet gear bearing clearance.

### 2.2. Sun Gear Tooth Crack Modeling

Sun gear tooth crack is a common tooth fault in the planetary gearbox, which could result in gearbox failures without early detection. If a planetary gearbox is damaged by tooth crack, it causes economic loss or catastrophic accidents. Therefore, this study focuses on the sun gear tooth crack, and the considered crack level is ranging from 0% to 50% with an increment of 0.5%. So, there are 101 crack cases involved. The 0% crack level means there is no tooth crack, and the 50% level corresponds to the crack length of 3.90 mm.

**Figure 5:**
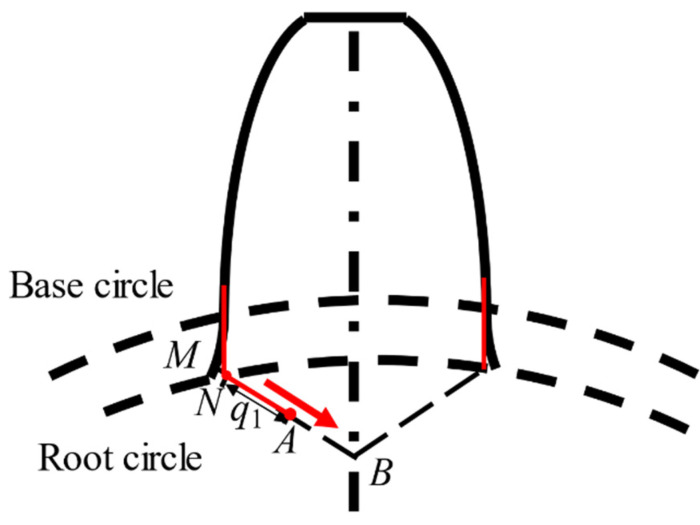
The schematic of a sun gear tooth with root fillet crack [20].

**Figure 6:**
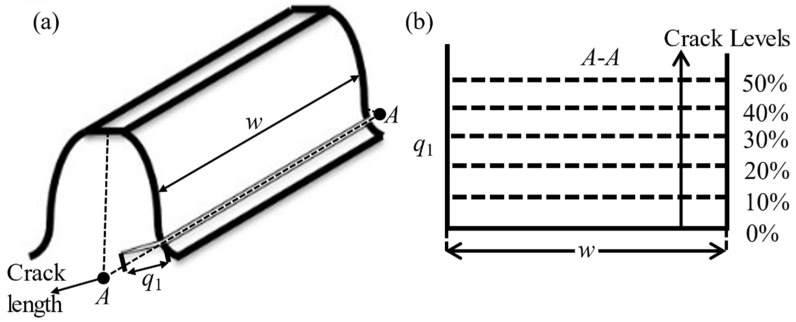
Length and width propagation (**a**) model, (**b**) the related scenario [31].

In this paper, the root circle of sun gear is lower than its base circle, and thus the tooth crack modeling (Figure 5) is the same as that in Reference [20]. In this figure, the *q*_1_ is the crack length, and the crack initiation point is point *N* on the root circle. Then, the crack is simulated with a straight-line growth from point *N* to point *A*, as shown in Figure 5. The extreme crack length is that the tooth crack propagates along the growth line from initiation point *N* to the endpoint *B*, which corresponds to the 50% crack level. Furthermore, the line segment *MN* is interpreted as the initial notch which is not considered as crack length [20]. Figure 6 shows the length and width propagation [31]. In this figure, *w* denotes the tooth face width and *q*_1_ is the crack length. When the length *q*_1_ increases, the width *w* always remains the same length *A*-*A* during the growth of the crack level. In this study, we have assumed that the crack thickness is about zero, which is neglected. This cracked tooth model is used in another investigation [29] as well.

Wu et al. [32] researched the effect of tooth crack, showing that the influence caused by the crack is observable when the crack level grows. Pandya and Parey [33] pointed out that the vibration level changes drastically for an advanced crack (more than 40%), and the accuracy of the mesh stiffness generated by the potential energy model decreases through the crack level growth. Thus, this paper focuses on the effect of bearing clearance for a planetary gearbox with a relatively low sun gear tooth crack level, ranging from 0% to 50%. Furthermore, 6 different crack levels are used to analyze the effect of sun gear tooth crack on a planetary gearbox, and these levels include healthy level (0%), low crack levels (10%, 20%, 30%, and 40%), and half-tooth level (50%). The correspondence between crack levels and crack length is shown in Table 3.

Based on the real planetary gearbox and the sun gear crack model introduced above, the analytical method (AM) reported in Reference [29] is used to model the sun gear tooth crack in this study.

### 2.3. Bearing Clearance Modeling

**Figure 7:**
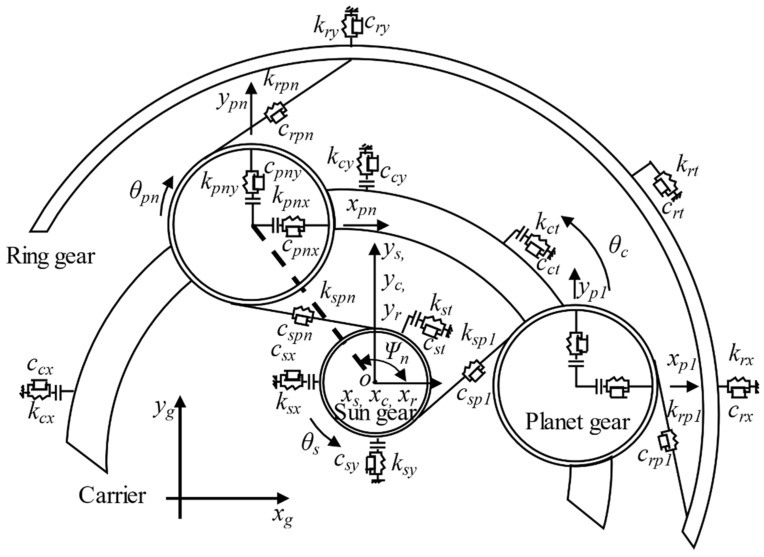
Lumped parameter model of planetary gear set [29].

According to previous studies, all the bearings have their own designed clearances [14,27]. The coordinate system of planet gears has a different origin point from the coordinate systems for the carrier and planet gears in Guo and Parker’s work [24]. This paper uses the rotating coordinate systems with the same origin point, *o*, shown in Figure 7 to describe gear motions, which are developed based on those in References [24,28,29]. Thus, this paper needs to modify the equations of bearing clearance model described in the reference [24]. To be specific, the relative displacement between carrier and planet gear is equal to ${\left[{\left(x_{c}\cos\Psi_{j}+y_{c}\sin\Psi_{j}-\xi_{j}\right)}^{2}+{\left(-x_{c}\sin\Psi_{j}+y_{c}\cos\Psi_{j}+u_{c}-\eta_{j}\right)}^{2}\right]}^{1/2}$ in Reference [24]. $x_{c}$ and $y_{c}$ denote the translational displacement for the carrier, and the origin point of their coordinate system is the center of the planetary gearbox (point *o*). $\xi_{j}$ and $\eta_{j}$ are the radial and tangential displacements of the planets, and the origin point of their coordinate system is the center of the planet gear. In this paper, point *o* is the origin point for all coordinate systems, which is the same as Reference [29], and thus the relative displacement between carrier and planet gear can be modified as Equation (3). $x_{pn}$ and $y_{pn}$ are the translational displacement of planet gear in the *x*-axis and *y*-axis [29].

Based on the test rig (Figure 3), the lumped parameter model of Stage 2 can be shown, as in Figure 7. The subscripts *r*, *c*, *p* and *s* represent ring gear, carrier, planet gear, and sun gear, respectively. *x* and *y* indicate two planar degrees of freedom, and the *θ* is the rotational degrees of freedom. This means that one gear has three directions: *x*, *y*, and *θ*. In this paper, there are four planet gears, one sun gear, one carrier, and one ring gear, and thus the total degrees of freedom are 21. Thus, 21 motion equations are used to simulate the dynamic modeling, and the details about these equations are presented below.

The equations of carrier-planet bearing force (*F_cpnx_* and *F_cpny_*) are different from Reference [29] after considering the planet gear bearing clearance. In this study, the equations for computing these forces considering bearing clearance are developed based on those presented in References [24,29]. *µ* and (δ−Δ) are used to calculate the effect of bearing clearance [24]. This study uses these variables (μ, δ, and Δ) to modify the bearing force equations reported in Reference [29]. The modified part $\delta_{cpn}-\Delta$*_p_* is used to caculate the relative displacment between the carrier and *n*th planet gear. Then, $\vartheta_{cpn}$ is used to transfer the direction of displacement to *x*- or *y*-direction. Eventually, the planet bearing force can be calculated by the following modified equations:

(1) $$F_{cpnx}=\mu_{cpn}k_{pnx}\left(\delta_{cpn}-\Delta_{p}\right)\cos\left(\vartheta_{cpn}\right)+\mu_{cpn}c_{pnx}\left({\dot{x}}_{pn}-{\dot{x}}_{c}\right)$$

(2) $$F_{cpny}=\mu_{cpn}k_{pny}\left(\delta_{cpn}-\Delta_{p}\right)\sin\left(\vartheta_{cpn}\right)+\mu_{cpn}c_{pny}\left({\dot{y}}_{pn}-{\dot{y}}_{c}\right)$$

where *k* and *c* are bearing stiffness and damping. Δ and *δ* are bearing clearance and gear’s displacement. Subscripts *pn* and *c* denote the *n*-th planet gear and carrier. Furthermore, subscripts *x* and *y* are two coordinate axes perpendicular to each other in the rotating coordinate system. For example, *c_pnx_* and *c_pny_* are the bearing damping in the *x*- and *y*-direction. Based on the same coordinate system used in carrier and planet gears, their displacement can be calculated directly in the same axis, such as $x_{pn}-x_{c}$ in the *x*-direction. Thus, the equation of relative displacement *δ_cpn_* is:

(3) $$\delta_{cpn}=\sqrt{{\left(x_{pn}-x_{c}\right)}^{2}+{\left(y_{pn}-y_{c}\right)}^{2}}$$

The contact angle, *ϑ*, between a planet and carrier is calculated as follows:

(4) $$\vartheta_{cpn}=\{\begin{array}{ccc} \tan^{-1}\left(\frac{y_{pn}-y_{c}}{x_{pn}-x_{c}}\right) & \mathrm{if} & x_{pn}>x_{c}\\ \frac{\pi }{2} & \mathrm{if} & x_{pn}=x_{c},y_{pn}>y_{c}\\ -\frac{\pi }{2} & \mathrm{if} & x_{pn}=x_{c},y_{pn}<y_{c}\\ \pi +\tan^{-1}\left(\frac{y_{pn}-y_{c}}{x_{pn}-x_{c}}\right) & \mathrm{if} & x_{pn}<x_{c} \end{array}$$

The meaning of *x* and *y* are mentioned above. *µ_cpn_* can be subsequently determined as follows [24]:

(5) $$\mu_{cpn}=\{\begin{array}{c} \begin{array}{ccc} 1, & if & \delta_{cpn}>\Delta_{p} \end{array}\\ \begin{array}{ccc} 0, & if & \delta_{cpn}<\Delta_{p} \end{array} \end{array}$$

From Equation (5), it is noted that if $\delta_{cpn}$ is smaller than the Δ*_p_*, μ is 0, which means there is no bearing force (*F_cpnx_* = 0). Otherwise, μ is 1. Similar to the modification between Equations (1) and (5), the motion equations of dynamic modeling adopted in this paper are modified versions of those reported in Reference [29].

### 2.4. Motion Equations

The motion equations of the sun gear are modified based on those presented in References [24,28,29]. The modified bearing forces due to bearing clearance are given in Equations (1) and (2). Other modified motion equations are:

(6) $$\begin{array}{c} m_{s}{\ddot{x}}_{s}+\mu_{s}c_{sx}{\dot{x}}_{s}+\mu_{s}k_{sx}\left(\delta_{s}-\Delta_{s}\right)\cos\left(\vartheta_{s}\right)+\sum_{n}F_{spn}\cos\Psi_{sn}=m_{s}x_{s}\Omega^{2}+2m_{s}{\dot{y}}_{s}\Omega +m_{s}y_{s}\dot{\Omega }\\ m_{s}{\ddot{y}}_{s}+\mu_{s}c_{sy}{\dot{y}}_{s}+\mu_{s}k_{sy}\left(\delta_{s}-\Delta_{s}\right)\sin\left(\vartheta_{s}\right)+\sum_{n}F_{spn}\sin\Psi_{sn}=m_{s}y_{s}\Omega^{2}-2m_{s}{\dot{x}}_{s}\Omega -m_{s}x_{s}\dot{\Omega }\\ \left(J_{s}/r_{s}\right){\ddot{\theta }}_{s}+\sum_{n}F_{spn}=T_{i}/r_{s} \end{array}$$

where Δ is the bearing clearance, *r* is the gear radius, and *θ* is the rotational displacement. The subscript *s* refers to the sun gear. Then, the *T_i_* and *J* denote the input torque and the mass moment of inertia, respectively. Furthermore, coefficient *δ_s_* is given as:

(7) $$\delta_{s}=\sqrt{x_{s}{}^{2}+y_{s}{}^{2}}$$

The coefficients *ϑ_s_* and *µ_s_* are obtained by replacing the subscript *cpn* of their equivalents in Equations (3) and (4) by *s*, respectively. The dynamic force, *F_spn_*, between the sun and *n*-th planet gear can be found in Reference [29].

For the fixed ring gear, there is no ring bearing considered, and thus the expression of motion equations for the ring gear is same as that in Reference [29]. The expression of motion equations for the planet gears is also the same as those presented in Reference [29]. This paper only presents the modified parts, such as the $F_{cpnx}$ and $F_{cpny}$ in Equations (1) and (2). For the carrier, the motion equations are modified based on the corresponding equations in References [24,28,29]. By incorporating the modified bearing force equations, the modified motion equations are given as follows:

(8) $$\begin{array}{c} m_{c}{\ddot{x}}_{c}+\mu_{s}c_{cx}{\dot{x}}_{c}+\mu_{s}k_{cx}\left(\delta_{c}-\Delta_{c}\right)\cos\left(\vartheta_{c}\right)-\sum_{n}F_{cpnx}=m_{c}x_{c}\Omega^{2}+2m_{c}{\dot{y}}_{c}\Omega +m_{c}y_{c}\dot{\Omega }\\ m_{c}{\ddot{y}}_{c}+\mu_{s}c_{cy}{\dot{y}}_{c}+\mu_{s}k_{cy}\left(\delta_{c}-\Delta_{c}\right)\sin\left(\vartheta_{c}\right)-\sum_{n}F_{cpny}=m_{c}y_{c}\Omega^{2}-2m_{c}{\dot{x}}_{c}\Omega -m_{c}x_{c}\dot{\Omega }\\ \left(J_{c}/r_{c}\right){\ddot{\theta }}_{c}+\sum_{n}F_{cpnx}\sin\Psi_{n}-\sum_{n}F_{cpny}\cos\Psi_{n}=T_{o}/r_{c}\;\; \end{array}$$

Similar to the Equation (6), Δ is the bearing clearance, *r* is the radius, and *θ* is the rotational displacement. Subscript *c* refers to the carrier. Furthermore, *T_o_* is the output torque, due to the carrier being the output. The *δ_c_* is given as:

(9) $$\delta_{c}=\sqrt{x_{c}{}^{2}+y_{c}{}^{2}}$$

The coefficients *ϑ_c_* and *µ_c_* are obtained by replacing the subscript *cpn* of their equivalents in Equations (3) and (4) by *c*, respectively.

In this paper, the gear rotates with a constant speed, and thus the acceleration in *θ* direction is 0 for each component in the planetary gearbox, including the sun gear, carrier, four planet gears, and ring gear.

As mentioned above, the coordinate of the motion equations is fixed with the carrier, which means these coordinates rotate with the carrier. However, the fixed reference system is used to analyze the vibration signals. Therefore, we can use the coordinate transform equations in Equation (10) to relate these two systems:

(10) $$\left[\begin{array}{c} x_{g}\\ y_{g} \end{array}\right]=\left[\begin{array}{cc} \cos\left(t\Omega_{c}\right) & -\sin\left(t\Omega_{c}\right)\\ \sin\left(t\Omega_{c}\right) & \cos\left(t\Omega_{c}\right) \end{array}\right]\left[\begin{array}{c} x\\ y \end{array}\right]$$

where Ω*_c_* is the rotating speed of the carrier, *t* is time, *x* and *y* denote the rotating coordinate system, and *x_g_* and *y_g_* denote the fixed rotating coordinate system. The transmission path is not considered in this study. Thus, we have assumed that there is no attenuation of the vibration signal in the planetary gearbox. Liu et al. [30] presented an equation for the vibration responses of the planetary gearbox. In this paper, the transmission path effects are ignored, which means that the coefficients characterizing the transmission path effects presented in Reference [30] can be set to be 1. Therefore, the planetary gearbox signal model for the signal in *x* and *y* directions are simplified as follows:

(11) $$x_{signal}=x_{sg}+\overset{4}{\underset{n=1}{\sum }}x_{png}+x_{cg}+x_{rg}$$

(12) $$y_{signal}=y_{sg}+\overset{4}{\underset{n=1}{\sum }}y_{png}+y_{cg}+y_{rg}$$

where *x_signal_* and *y_signal_* denote the vibration signal for the whole planetary gearbox in *x_g_* and *y_g_* direction, and subscript *pn* denotes the *n*th planet, respectively.

The dynamic response of sun gear with the rotating coordinate system (*x_c_*) was presented and analyzed in Reference [14], and thus this paper focuses on the sum vibration signal (*x_signal_*), to be discussed in the next section.

## 3. Results and Discussions

**Figure 8:**
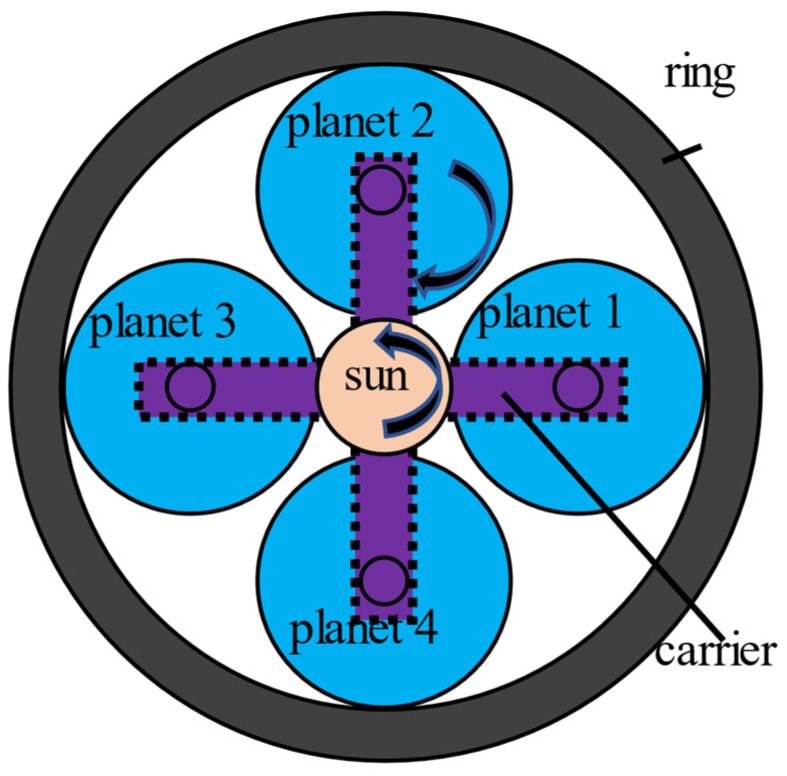
Planetary gearbox with four planet gears.

As mentioned in Section 2, the Stage 2 planetary gearbox has four planet gears, and the numbering of each planet gear is shown in Figure 8. The sun gear is the input rotating in the counterclockwise direction, while the directions of planet gears are clockwise.

**Figure 9:**
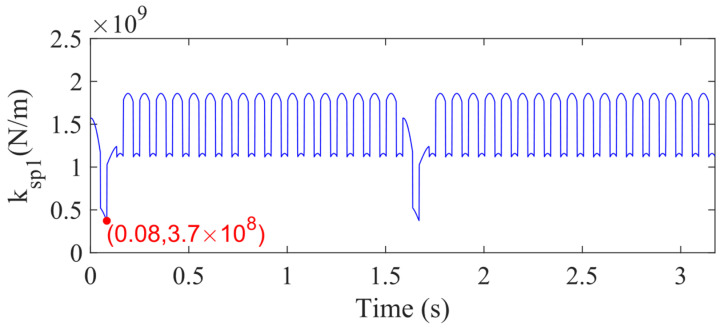
Meshing stiffness of sun-planet 1 with 50% sun gear tooth crack.

Based on the sun gear tooth crack model, we can evaluate the mesh stiffness (*k*). Figure 9 shows the sun-planet mesh stiffness for 50% sun gear tooth crack level, which is used to calculate the planetary gearbox dynamic responses. The *k_sp_*_1_ denotes the mesh stiffness for the mesh pair consisting of the sun gear and the planet 1. From Figure 9, it is found that the sun gear tooth crack decreases the mesh stiffness to 3.7 × 10^8^ N/m. The minimum values of the meshing stiffness for mesh pairs sun-planet 2, 3, and 4 are all 3.7 × 10^8^ N/m. According to the mesh stiffness, motion equations, and the parameters of the planetary gear set listed in Table 4, the vibration response of the planetary gearbox can be calculated through the ODE15S function in MATLAB.

**Figure 10:**
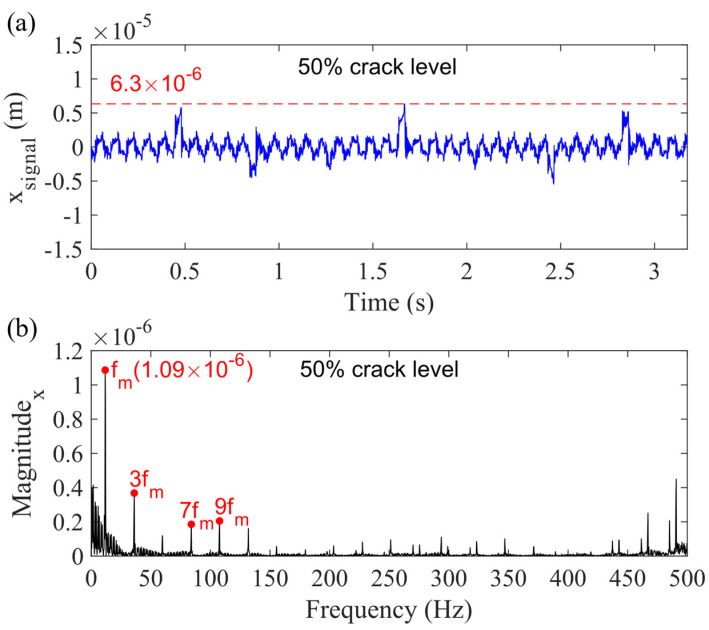
Sum vibration response for 50% sun gear crack level in *x_g_*-direction: (**a**) time domain waveform and (**b**) frequency spectrum.

The vibration response of each gear can be obtained by solving the motion equations. If all the bearing clearance is 0 mm, which means the model does not consider the bearing clearance, the obtained responses are the same as those in Reference [29]. Figure 10 shows the planetary gearbox vibration response in the x_g_-direction for this case. Figure 10a is the vibration response in the time domain, and (b) is its frequency spectrum. The dashed line marking the highest value of vibration response is 6.3 × 10^−6^ m, while the magnitude of fundamental gear mesh frequency (11.97 Hz) is 1.09 × 10^−6^. By comparing the vibration responses for Case 1 and those for other cases, the effect of different types of bearing-clearance on planetary gearbox vibration responses can be studied.

### 3.1. Single Type of Bearing Clearance

**Figure 11:**
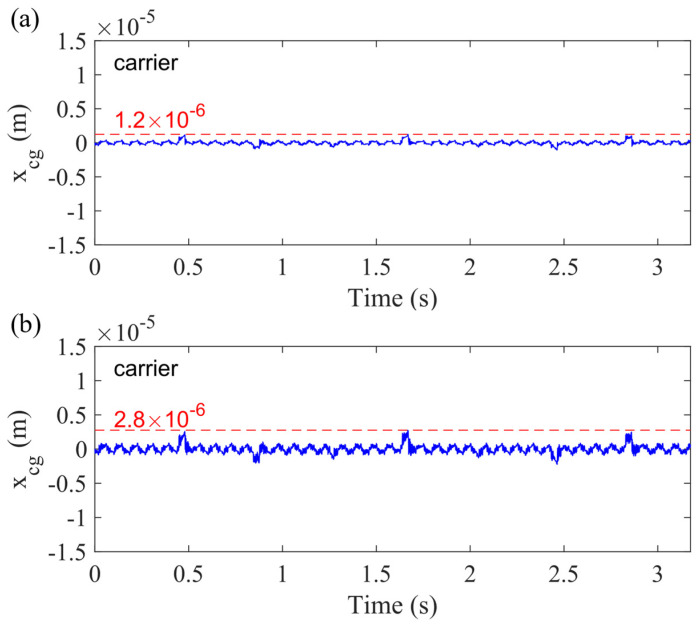
Vibration response of carrier for the 50% sun gear crack level for (**a**) Case 1 and (**b**) Case 2, both in *x_g_*-direction.

In this section, we only consider one type of bearing clearance. The carrier bearing clearance (0.080 mm), analyzed in Case 2. Figure 11 presents the vibration of the carrier in the *x*_*g*_-direction fixed with the ground, and the sun gear crack level is 50%. Figure 11a is the vibration response without any bearing clearance (Case 1), and (b) is the vibration signal for the scenario only considering the carrier bearing clearance (Case 2). Comparing these two plots, we can find that the carrier bearing clearance increases the vibration of the carrier from 1.2 × 10^−6^ to 2.8 × 10^−6^ m.

**Figure 12:**
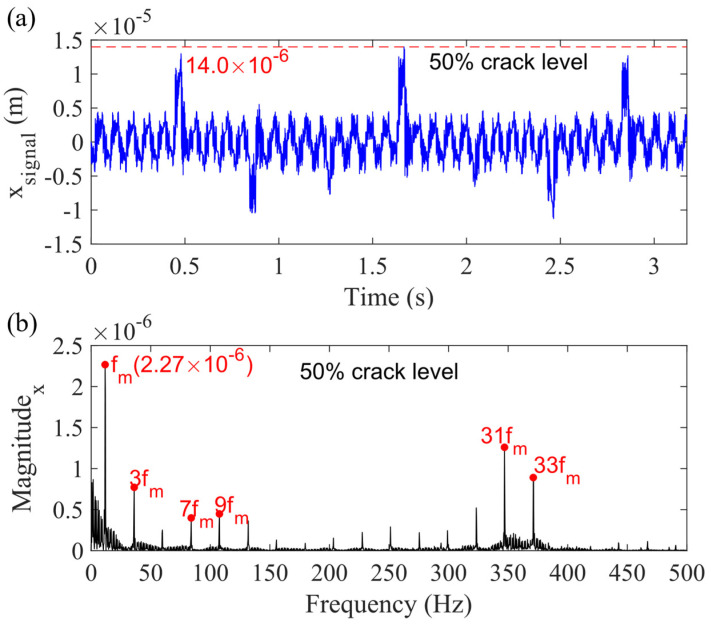
Sum vibration response for 50% sun gear crack level for Case 2 in (**a**) time domain and (**b**) frequency domain, in *x_g_*-direction.

Other vibration responses for sun gear, planet gears, and ring gear can also be generated by the motion equations. Then, the sum vibration signal for the planetary gearbox can be obtained by adding these signals together through the Equations (10)–(12). Figure 12a presents the sum vibration signal for the case considering the carrier bearing clearance for the 50% sun gear tooth crack level, while (b) is the spectrum. Comparing Figure 12 to Figure 10, it is found that the highest value of the signal is significantly increased from 6.3 × 10^−6^ to 14.0 × 10^−6^ m, and the magnitude of the fundamental gear mesh frequency (*f_m_*) becomes 2.27 × 10^−6^ m. Furthermore, a harmonic cluster is also emerging in the frequency range from 350 to 400 Hz.

**Figure 13:**
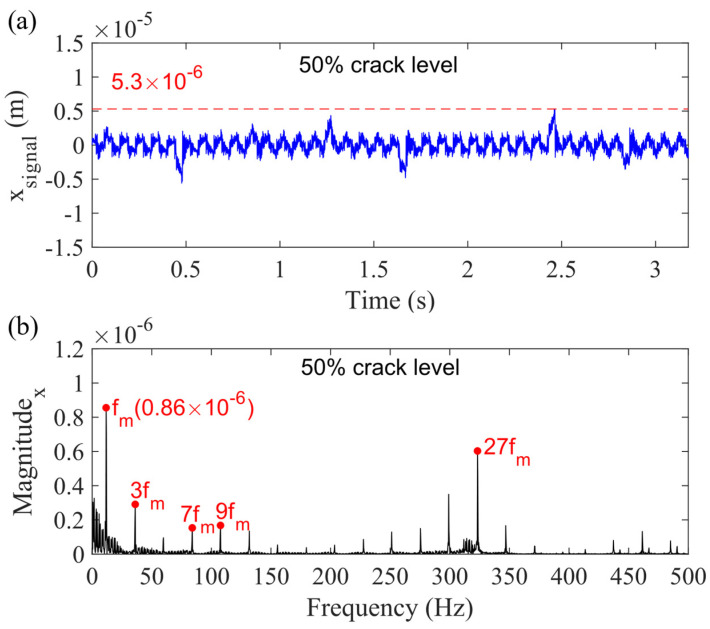
Sum vibration response with 50% sun gear crack level for Case 3 in (**a**) time domain and (**b**) frequency domain in *x_g_*-direction.

Case 3, considering the sun gear bearing clearance (0.080 mm), however, reduces the sum vibration signal. The phenomenon is discussed in Case 5. Figure 13a presents the sum vibration signal considering the sun gear bearing clearance with 50% sun gear tooth crack, while (b) is the spectrum. Compared with Figure 10, the highest value of the signal is reduced from 6.3 × 10^−6^ to 5.3 × 10^−6^ m, and the magnitude of mesh frequency (*f_m_*) is decreased to 0.86 × 10^−6^. Furthermore, a new frequency cluster emerges around 27 *f_m_*, which means the 27th harmonic of *f_m_*.

**Figure 14:**
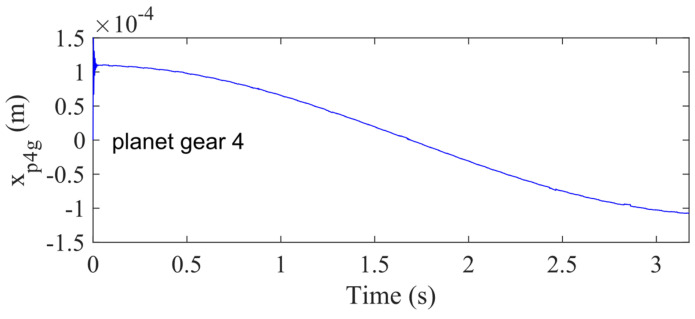
Vibration response in *x_g_*-direction for the fourth planet gear with 50% sun gear crack level for Case 4.

Case 4 shows that the planet gear bearing clearance is much smaller than its displacement. Figure 14 shows the displacement of planet 4, and it is easy to find that the highest displacement is around 0.11 mm. Then, the other planet gears’ signal is similar to Figure 14. Furthermore, the planet gear bearing clearance is only 0.035 mm, which is much smaller than its displacement.

**Figure 15:**
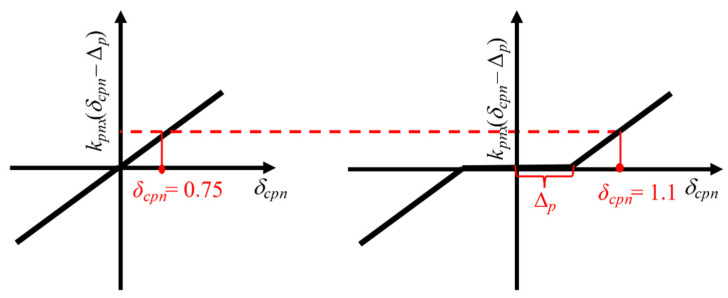
The schematic diagram for the *k_pnx_* (*δ_cpn_* − Δ*_p_*).

From Equation (1), the planet gear bearing clearance can affect the bearing force, *F_cpnx_* and *F_cpny_*. Figure 15 shows the schematic diagram for this equation. Although the planet gear bearing clearance (Δ*_p_*) increases *δ_cpn_*, the final value marked by the dashed line is the same. Therefore, the effect of planet gear bearing clearance could be neglected.

**Figure 16:**
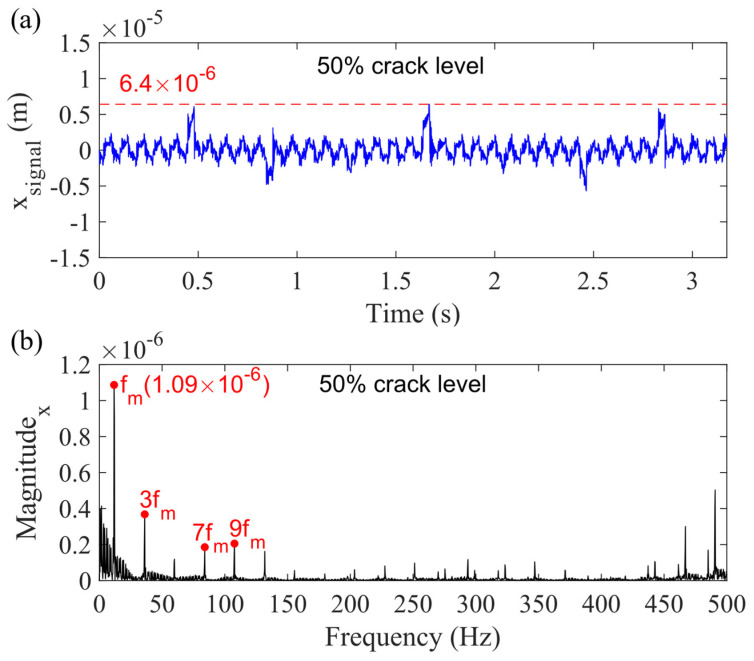
Sum vibration response with 50% sun gear crack level for Case 4 in (**a**) time domain and (**b**) frequency domain in *x_g_*-direction.

The vibration response for Case 4 is shown in Figure 16. Figure 16a presents the sum vibration signal considering the planet gear bearing clearance with 50% sun gear tooth crack, while Figure 16b shows the spectrum. Compared with Figure 10, the highest value of the signal is changed from 6.3 × 10^−6^ to 6.4 × 10^−6^ m with a very small increase, and the magnitude of mesh frequency (*f_m_*) is the same as Figure 10b.

In conclusion, the carrier and sun gear bearing clearance can affect the planetary gearbox vibration response, while the effect of the planet gear bearing clearance on the vibration responses is too small to be considered in the dynamic modeling.

### 3.2. Combinations of Multiple Bearing Clearance Types

This section considers the combinations of bearing clearance types, including Case 5 and 6.

**Figure 17:**
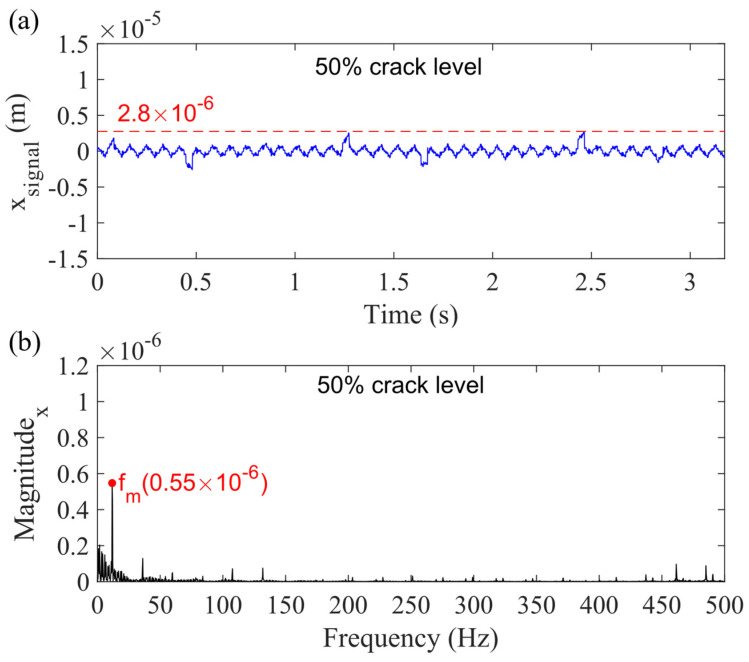
Sum vibration response with 50% sun gear crack level for Case 5 in (**a**) time domain and (**b**) frequency domain in *x_g_*-direction.

Case 5 is the simulated response considering the combination of sun gear bearing and carrier bearing clearances. The bearing clearance Δ_c_ of the carrier is determined to be 0.08 mm, and the sun gear bearing clearance Δ_s_ is determined to be 0.08 mm, (i.e., the minimum values in normal scenarios following the standards of Timken (North Canto, OH, USA) and NTN (Osaka, Japan)). The obtained vibration response of the planetary gearbox is shown in Figure 17, where the dashed line shows the highest displacement in the *x_g_*-direction for the 50% crack level. The *x_signal_* denotes the signal in the *x_g_*-direction. Figure 17b shows that the magnitude of gear mesh frequency (*f_m_*) is reduced from 1.09 × 10^−6^ to 0.55 × 10^−6^. The frequency contents are significantly decreased, especially from 450 to 500 Hz. Furthermore, compared with Figure 10a, two interesting phenomena are found. First, the highest displacement decreases from 6.3 × 10^−6^ to 2.8 × 10^−6^ m rather than increases, and the reduction trend is similar to Case 3’s trend. This paper focuses on the displacements of the sun gear and planet gear to discuss this phenomenon.

**Figure 18:**
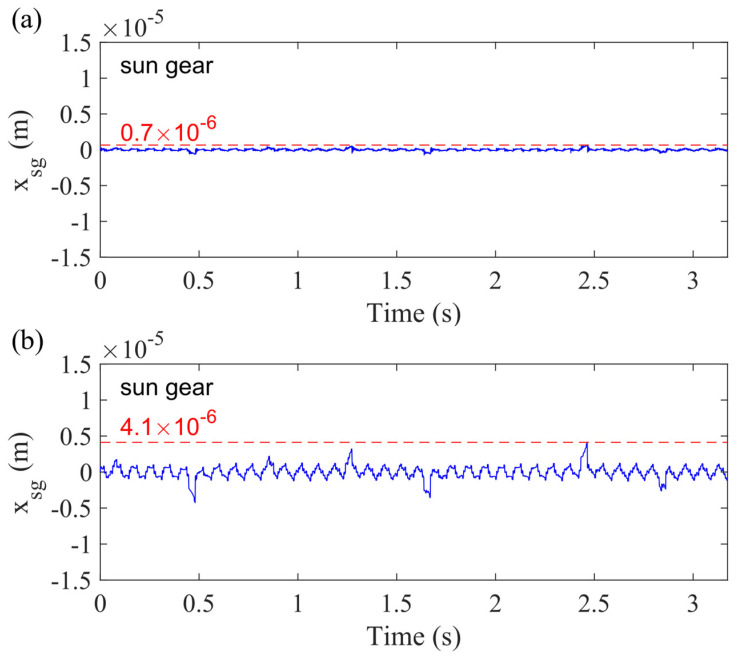
Vibration response of sun gear with 50% sun gear crack level for (**a**) Case 1 and (**b**) Case 5 in *x_g_*-direction.

Figure 18 shows the vibration signal of the sun gear with 50% sun gear crack. It is obvious to find that the sun gear and carrier bearing clearance increase the displacement of the sun gear from 0.7 × 10^−6^ to 4.1 × 10^−6^. Figure 18a is the vibration response of the sun gear without any bearing clearance (Case 1), and Figure 18b is the vibration responses of the sun gear considering the sun gear and carrier bearing clearance (Case 5). *x_sg_* denotes the displacement of sun gear in the *x_g_*-direction.

**Figure 19:**
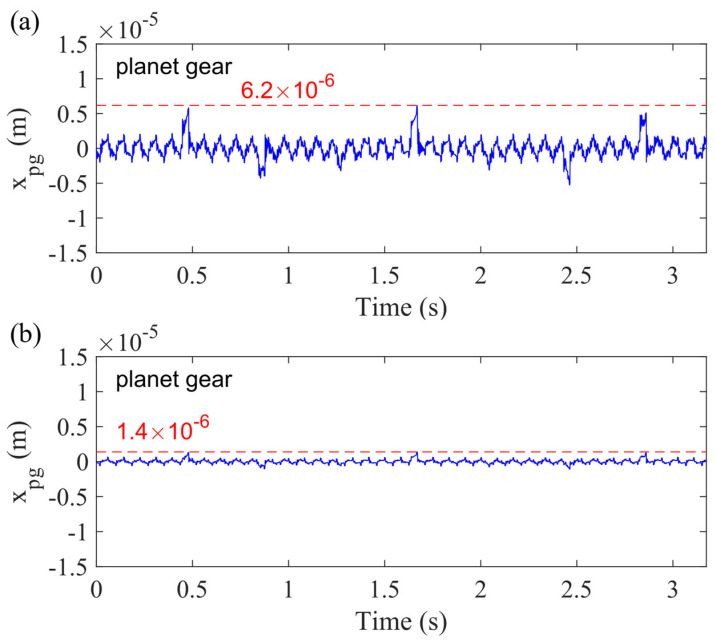
Vibration response of planet gear with 50% sun gear crack level considering (**a**) no and (**b**) sun gear and carrier bearing clearance in *x_g_*-direction.

However, the sun gear and carrier bearing clearance significantly reduce the displacement of planet gear, as shown in Figure 19, where the dashed line shows the highest displacement in the *x_g_*-direction for the case of the 50% crack level. It is obvious to find that the sun gear and carrier bearing clearance decreases the highest value of the sum displacement for four planet gears, from 6.2 × 10^−6^ to 1.4 × 10^−6^. Figure 19a is the vibration signal of planet gear without any bearing clearance (Case 1), and Figure 19b is the vibration signal of planet gear considering the sun gear and carrier bearing clearance (Case 5). *x_pg_* is the sum displacement for the four planet gears in the *x_g_*-direction. Therefore, when adding the displacement of the sun gear and planet gears together, the sum vibration response in Case 1 (Figure 10a) is higher than the sum signal in Case 5 (Figure 17a).

The second interesting phenomenon is that the direction of impulse caused by crack is opposite between Figure 10a and Figure 17a. From Figure 18 and Figure 19, it is obvious to find that the directions of crack-caused impulses are opposite. For example, around 0.5 s, the direction of the vibration signal for the sun gear is downward (negative value), while the vibration direction is upward (positive value) for the planet gear. Then, the displacement of sun gear in Figure 18b is higher than the movement of planet gear in Figure 19b. Therefore, the direction of sum vibration is the same as that of the sun gear’s movement after considering the sun gear and carrier bearing clearance. In conclusion, the bearing clearance does not only affect the displacement, but also changes the direction of crack-caused impulse for the sum vibration response.

**Figure 20:**
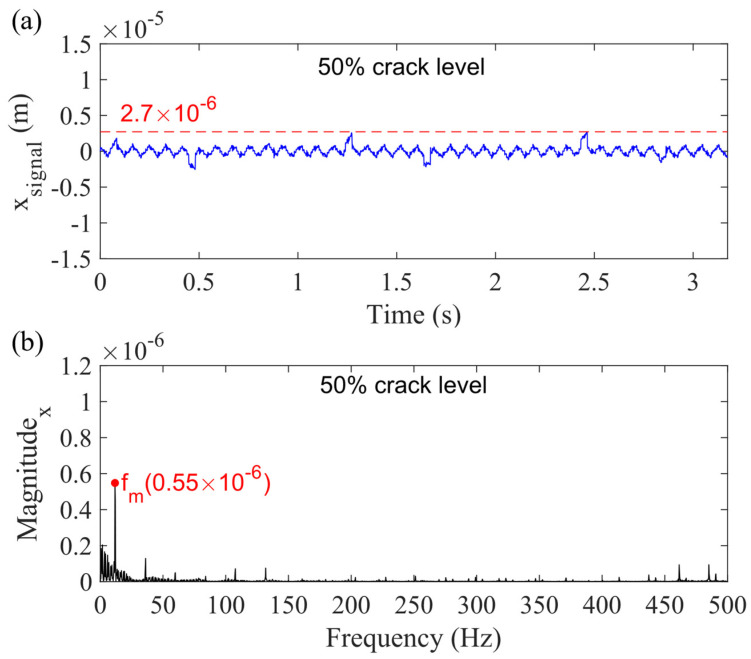
Sum vibration response with 50% sun gear crack level for Case 6 in (**a**) time domain and (**b**) frequency domain in *x_g_*-direction.

As mentioned in Case 4, the planet gear bearing clearance could not affect the vibration response. This conclusion still holds in Case 6. Figure 20 shows the simulation vibration signal for the planetary gearbox with 50% sun gear tooth crack in the *x_g_*-direction. Figure 20a presents the sum vibration signal considering all types of bearing clearance, and Figure 20b shows the spectrum. Compared with Figure 17, it is obvious to find that the highest value changes from 2.6 × 10^−6^ to 2.7 × 10^−6^ m.

As mentioned above, this paper simulated the vibration responses for the cases of 0%, 10%, 20%, 30%, 40% and 50% sun gear crack levels. Table 5 shows the maximum value in the time domain for each case with different crack levels. The maximum values for Case 1 are close to those for Case 4, and the values for Case 5 are close to those for Case 6. This situation points out that the effect of planet gear bearing clearance could be neglected in the dynamic modeling. Thus, Case 5 with carrier and sun gear bearing clearance should be closer to the real system, because this case considers two effective bearing clearances (carrier and sun gear).

**Figure 21:**
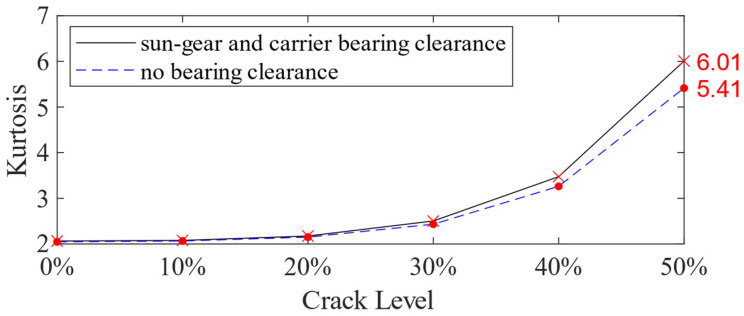
Kurtosis of the vibration signal for the planetary gearbox with 0%, 10%, 20%, 30%, 40% and 50% crack levels.

The Kurtosis curve is used to analyze the vibration responses for Case 1 and Case 5. Figure 21 illustrates the Kurtosis index, and the horizontal axis represents different crack levels, from 0% to 50%. The solid line depicts the results obtained by the dynamic simulation considering the sun gear and carrier bearing clearance (Case 5), while the dashed line represents those without considering the bearing clearance (Case 1). From the results for these two cases in Table 5 and Figure 21, although the combination of sun-gear and carrier bearing clearance reduces the maximum value in the time domain, it increases the kurtosis, especially when the crack level is greater than 30%. The increase in the kurtosis could also benefit early fault detection.

## 4. Conclusions

The dynamic modeling for a planetary gearbox with the bearing clearance and sun gear tooth crack are simultaneously focused on in this paper. The main contributions of this study include the following: (1) development of the dynamic model for a planetary gearbox with both bearing clearance and sun gear tooth crack, (2) establishment of the motion equations for the planetary gearbox test-rig used in the Reliability Research Lab at the University of Alberta, and (3) investigation of the dynamic signals of different sun-gear tooth crack levels. The two major assumptions are the constant bearing clearance and no interaction between Stages 1 and 2 gearboxes.

The following observations can be obtained: (1) not all bearing clearance can influence the vibration responses, (2) the planet gear bearing clearance’s influence can be neglected, (3) the bearing clearances does not only affect the magnitude of the vibration signal but also changes the direction of crack-caused impulse, and (4) the combination of effective bearing clearance, such as sun gear and carrier, can increase the kurtosis with the increase of crack levels. This paper is extended from the conference paper presented at APARM 2020 [14]. 

## Figures and Tables

**Table 1:** The number of teeth and reduction ratio of each gearbox in the test rig.

	Bevel Gearbox		Stage 1			Stage 2		
	Input Gear	Output Gear	Ring Gear	Sun Gear	Planet Gear	Ring Gear	Sun Gear	Planet Gear
No. of teeth	18	72	152	28	62	81	19	31
Reduction ratio	4.000		6.429			5.263		

**Table 2:** Parameters of two carrier bearings and planet gear bearings for the two-stage planetary gearboxes.

Location	Description	Inner Race Diameters (mm)	Outer Race Diameters (mm)	Width (mm)	No. of Rollers
Sun gear bearing	Timken 42,584/42,381	96.838	148.4	28.58	26
Carrier bearing	Timken 42,584/42,381	96.838	148.4	28.58	26
Planet gear bearing	NTN 4T-32005X	25	47	15	19

**Table 3:** Crack levels and the corresponding length.

**Crack Levels**	0%	10%	20%	30%	40%	50%
**Crack Length (mm)**	0	0.78	1.56	2.34	3.12	3.90

**Table 4:** Parameters of the modeled planetary gearbox [29].

Parameters	Sun Gear	Planet Gear	Ring Gear
No. of teeth	19	31	81
Module (mm)	3.2	3.2	3.2
Pressure Angle (°)	20	20	20
Mass (kg)	0.700	1.822	5.982
Face width (mm)	38.1	38.1	38.1
Young’s Modulus (GPa)	2.068 × 10^5^	2.068 × 10^5^	2.068 × 10^5^
Poisson’s ratio	0.3	0.3	0.3
Base circle radius (mm)	28.3	46.2	120.8
Bearing stiffness (N·m)	*k_sx_* = *k_sy_* = *k_rx_* = *k_ry_* = *k_cx_* = *k_cy_* = *k_pnx_* = *k_pny_* = 1.0 × 10^8^		
Bearing damping (N·s/m)	*c_sx_* = *c_sy_* = *c_rx_* = *c_ry_* = *c_cx_* = *c_cy_* = *c_pnx_* = *c_pny_* = 1.5 × 10^3^		
Bearing clearance (mm)	Δ_c_ = Δ_s_ = 0.080, Δ_p_ = 0.035		

**Table 5:** The maximum value in the time domain for each case with different crack levels.

	0%	10%	20%	30%	40%	50%
Case 1 (no bearing clearance)	2.4 × 10^−6^ m	2.4 × 10^−6^ m	2.9 × 10^−6^ m	3.5 × 10^−6^ m	4.5 × 10^−6^ m	6.3 × 10^−6^ m
Case 2 (carrier bearing clearance)	4.9 × 10^−6^ m	5.3 × 10^−6^ m	6.2 × 10^−6^ m	7.8 × 10^−6^ m	10.0 × 10^−6^ m	14.0 × 10^−6^ m
Case 3 (sun gear bearing clearance)	2.3 × 10^−6^ m	2.4 × 10^−6^ m	2.8 × 10^−6^ m	3.2 × 10^−6^ m	4.0 × 10^−6^ m	5.3 × 10^−6^ m
Case 4 (planet gear bearing clearance)	2.4 × 10^−6^ m	2.7 × 10^−6^ m	3.0 × 10^−6^ m	3.7 × 10^−6^ m	4.7 × 10^−-6^ m	6.4 × 10^−6^ m
Case 5 (carrier and sun gear bearing clearance)	1.0 × 10^−6^ m	1.0 × 10^−6^ m	1.2 × 10^−6^ m	1.5 × 10^−6^ m	1.9 × 10^−6^ m	2.8 × 10^−6^ m
Case 6 (all bearing clearance)	1.0 × 10^−6^ m	1.1 × 10^−6^ m	1.3 × 10^−6^ m	1.5 × 10^−6^ m	1.9 × 10^−6^ m	2.7 × 10^−6^ m
